# Blood pressure and kidney size in term newborns with intrauterine growth restriction

**DOI:** 10.1590/S1516-31802007000200004

**Published:** 2007-03-04

**Authors:** Oscar Tadashi Matsuoka, Simone Shibao, Cléa Rodrigues Leone

**Keywords:** Blood pressure, Fetal growth retardation, Newborn infant, Kidney, Renin, Pressão arterial, Retardo do crescimento fetal, Recém-nascido, Rim, Renina

## Abstract

**CONTEXT AND OBJECTIVE:**

Low birth weight is associated with higher blood pressure in childhood and adulthood. The aim of this study was to investigate the influence of intrauterine growth restriction (IUGR) on newborn systolic blood pressure (SBP).

**DESIGN AND SETTING:**

Prospective comparative study at Neonatal and Intensive in Clinical Pediatrics Division, Maternity Hospital in Hospital das Clínicas, Faculdade de Medicina da Universidade de São Paulo.

**METHODS:**

35 newborns with IUGR and 35 without IUGR were compared. Healthy term newborns without malformations, with Apgar score at fifth minute > 6 were included. Birth weight, kidney weight/birth weight ratio, kidney weight (ultrasound scan), plasma renin activity (PRA) and SBP evolution were analyzed during the first month of life (on 1^st^, 3^rd^, 7^th^ and 30^th^ days).

**RESULTS:**

SBP evolution, kidney weight/birth weight ratio and PRA did not differ between the two groups. In newborns with IUGR, SBP presented positive correlations with birth weight (r = 0.387 p = 0.026) and BMI (r = 0.412 p = 0.017) on the 7^th^ day of life. Positive correlations with birth weight (r = 0.440 p = 0.01) and birth length (r = 0.386 p = 0.026) were also seen on the 30^th^ day. There was an inverse correlation on the 7^th^ day between SBP and kidney weight/birth weight ratio (r = -0.420 p = 0.014), but this did not persist to the end of the month.

**CONCLUSIONS:**

IUGR seems not to have any influence on SBP, PRA or kidney weight among term newborns during their first month of life.

## RESUMO

**CONTEXTO E OBJETIVO:**

O baixo peso ao nascimento está associado à pressão arterial mais elevada na criança e no adulto. O objetivo foi avaliar a influência do retardo do crescimento intra-uterino (RCIU) na pressão arterial sistólica de recém-nascidos (PAS).

**TIPO DE ESTUDO E LOCAL:**

Estudo prospectivo, realizado no berçário anexo à maternidade, Instituto da Criança, Hospital das Clínicas da Universidade de São Paulo.

**MÉTODOS:**

35 recém-nascidos (RN) com RCIU e 35 RN sem RCIU foram avaliados em um estudo prospectivo. Os critérios de inclusão foram: recém-nascidos no termo, saudáveis, sem malformações e índice de Apgar de quinto minuto > 6. Parâmetros antropométricos (peso ao nascimento, relação comprimento renal/peso ao nascimento), dimensões renais (pela ultrasonografia), dosagem da atividade da renina plasmática (ARP) e a evolução da PAS foram analisadas durante o primeiro mês de vida (no 1º, 3º, 7º e 30º dias de vida).

**RESULTADOS:**

A evolução da PAS, relação peso renal/peso ao nascimento e ARP não apresentaram diferença entre os dois grupos. Em recém-nascidos com CIUR, a correlação entre PAS e PN (r = 0,387 p = 0,026) e IMC (r = 0,412 p = 0,017) foi positiva no 7º dia de vida. No 30º dia de vida, a correlação com PN (r = 0,440 p = 0,01) e comprimento ao nascimento (r = 0,386 p = 0,026) também foi positiva. Houve correlação negativa entre a PAS do 7º dia e a relação peso do rim/peso ao nascimento (r = - 0,420 p = 0,014), porém, esta correlação não persistiu ao longo do primeiro mês de vida.

**CONCLUSÕES:**

RCIU parece não influenciar a PAS, a atividade de renina plasmática ou o peso renal em recém-nascidos no termo durante o primeiro mês de vida.

## INTRODUCTION

Epidemiological studies have documented an inverse relationship between birth weight and blood pressure levels in childhood and adulthood.^1^ According to the fetal origins hypothesis, birth weight (BW) is a marker for the quality of the intrauterine environment, which is responsible for programming blood pressure and other cardiovascular risks factors.^2^

There is evidence to suggest that uterine conditions may affect kidney size, which is a potential surrogate measurement for the number of nephrons. Various experimental models for intrauterine growth retardation have demonstrated that reduced kidney weight persists throughout the animal’s life, despite compensatory hypertrophy.^3-6^ In humans, low BW full-term infants have lower kidney weight and lower glomerular profile density than do infants with greater BW.^7^

Although a moderate deficiency in nephrons alone might not be expected to result in hypertension, several clinical observations and experimental studies have provided strong support for the notion that deficient numbers of nephrons predispose towards hypertension in later life. A direct relationship between total glomerular count and BW was demonstrated in autopsy kidneys;^8^ black subjects had smaller kidneys, attained higher blood pressures in response to sodium loading, and displayed reduced capacity to excrete sodium load;^9^ modest reductions in nephron count in rats, induced by gentamicin administration during gestation, were shown to be associated with glomerulosclerosis in maturity;^10^ maternal dietary protein restriction of 6% was found to be associated with mean systolic blood pressure of 159 mmHg in offspring at nine weeks, compared with 137 mmHg at nine weeks in offspring of rats that were fed normal protein intake.^11^

## OBJECTIVE

The present study was designed to investigate whether a significant relationship between intrauterine growth restriction (IUGR) and blood pressure is identifiable in newborns. Particularly, the possibility of relationships between anthropometrical parameters, kidney size, plasma renin activity and systolic blood pressure was explored.

## METHODS

A prospective study was conducted in the Nursery Annex of the Maternity Hospital, Neonatal and Intensive Clinical Pediatrics Division, Instituto da Criança, Hospital das Clínicas, Faculdade de Medicina da Universidade de São Paulo (HC-FMUSP), during the period from March 3, 2001, to May 5, 2003. The study was previously granted approval by the institution’s Ethics Committee for Research Project Analysis.

After obtaining written consent from the parents, newborns were selected at birth, in accordance with the inclusion criteria that they had to be healthy term newborns of gestational age ranging from ≥ 37 weeks to < 42 weeks, without malformations and with an Apgar score at the fifth minute > 6. Gestational age was determined from the mother’s last menstrual period, and was confirmed by a first-trimester scan. The exclusion criteria were: maternal use of antihypertensives, diuretics and corticosteroids during pregnancy, maternal addictions, maternal chronic arterial hypertension, genetic abnormalities and congenital infections in the newborns, multiple gestation, and respiratory, metabolic or infectious disorders in the newborns.

IUGR was defined as BW lower than the 10^th^ percentile of the intrauterine growth curves for gestational age for this hospital population, in association with the criteria of Kramer et al. for IUGR,^12^ in which the ratio between the observed BW and the mean BW (weight of 50^th^ percentile, WP50) (BW/WP50) for gestational age was lower than 0.85.

The newborns were divided into two groups: Group I (with IUGR) and Group II (without IUGR). The subjects for Group II were selected among those born immediately following the identification of each patient with IUGR.

Anthropometric variables at birth (weight, length and head circumference), indices (body mass index, ponderal index and arm circumference/head circumference ratio), gestational age confirmation, plasma renin activity (PRA) evaluation from the umbilical cord, kidney weight (evaluated by ultrasound, on the third day of life) and systolic blood pressure on the first and third days of life were obtained while the newborns were still in the maternity hospital.

At outpatient returns, anthropometric measurements and blood pressure were obtained on the 7^th^ and 30^th^ days, and a blood sample for PRA assay was collected on the 30^th^ day of life.

Blood was collected from the placental end of the umbilical vein following delivery of the baby and by venipuncture on the 30^th^ day of life. Four milliliters of the blood taken was added to a tube containing 0.5 ml of 0.3 mol disodium ethylene diamine tetraacetic acid (EDTA) and centrifuged at 4° C for 15 minutes. The plasma supernatant was decanted, divided into two aliquots and stored at -20° C until it was assayed for PRA.

A widely accepted non-invasive oscillometric monitor (Dixtal^®^) was used for the blood pressure measurements. These were performed on the right upper arm, which was stretched out parallel to the trunk, at heart level. The cuff was placed at the midpoint of the upper arm. The measurements were made while the newborn was in its own crib, in a wakeful state without crying, one hour after breast feeding, in the presence of the mother. The cuff sizes used followed the recommendations of the Second Task Force Report: the width of the inflatable part of the cuff bladder was 40% to 50% of the circumference of the arm.^13^ Three blood pressure readings were recorded on each measurement date, with 10-minute intervals between them. The final result taken was the mean of the values obtained.

Kidney weight was calculated using a portable grayscale ultrasound equipment (Toshiba Sonolayer^®^, model SSH-140 A/G) at newborn´s third day of life. This equipment is usually used at this nursery when any sonogram is required, for example: abdominal or transcranial sonogram. The examinations were performed by the same operator (a medical doctor working at the Radiology Institute), who was blinded to the infant’s status. The newborn was kept in ventral decubitus and the transducer was placed on the lumbar region to make the kidney measurements. The length, anteroposterior diameter and width of the kidney were measured for all the infants. The kidney volume was calculated using the following formula: kidney volume = 0.49 x length x width x anteroposterior diameter.^14^ The kidney weight was estimated from the volume found, using a conversion rate of 1 cm^3^ = 1 gram. The final result taken was the mean of the weights found for both kidneys. Kidneys present echogenicity that allows easy identification of their limits via ultrasound. Thus, measurement of this organ is a procedure that only presents a small degree of subjectivity.

Plasma renin activity (PRA) was measured indirectly, by means of the radioimmunoassay method, using the REN CT2^®^ device to quantify angiotensin I in human plasma. The examination consisted of quantifying the competence of the plasma for generating angiotensin I, under temperature and pH conditions relating to in vivo conditions. The results were expressed in nanograms of angiotensin I generated per milliliter per hour (ng/ml/h).

## STATISTICAL ANALYSIS

The sample size calculation was based on an alpha of 5% and a test power of 80%. The standard deviation for mean arterial pressure was taken to be 20 mmHg, with the aim of detecting a difference in mean pressure of at least 25%. Thus, a sample size of 35 newborns for each group was obtained.

The population was described by means of central tendency measurements (means) and dispersions (standard deviations), and also percentages.

Student’s t test was used to compare pairs of means. Whenever the assumptions for this test were not satisfied, its non-parametric equivalent (the Mann-Whitney test) was brought in. To evaluate continuous variables, one-way analysis of variance (ANOVA test) was used. To analyze arterial pressures in relation to anthropometric variables and the plasma renin activity, Pearson’s correlation test was used. The statistical significance level was set at 0.05.

## RESULTS

The characteristics of the newborns are presented in Table 1.

**Table 1:** Characteristics of the newborns at birth

Characteristics of the newborns	Group I with IUGR n = 35	Group II without IUGR n = 35
**Gender**		
Male	16 (45%)	22 (62.8%)*
**Gestational age (weeks)**	38.3 + 0.9	39.6 + 1.2*
**Type of delivery**		
Vaginal	16 (45.6%)	13 (37.1%)
Cesarean	19 (54.2%)	22 (62.8%)
**Race**		
White	18 (51.4%)	18 (51.4%)
Black	3 (8.5%)	3 (8.5%)
Other	14 (40.1%)	14 (40.1%)
**Diseases**		
Gestational hypertension	5 (14.2%)*	2 (5.7%)
Asthma	2 (5.7%)	2 (5.7%)
Sickle cell disease	2 (5.7%)	0
Others	5 (14.2%)	3 (8.5%)
**BW (grams)**	2247 + 170.4	3338 + 210.1*
**Length (cm)**	44.8 + 1.25	49.1 + 1.40*
**Head circumference (cm)**	32.1 + 0.9	34.8 + 0.8*
**BW/WP50**	0.75 + 0.05	1.02 + 0.08*
**AC/HC**	0.26 + 0.01	0.29 + 0.01*
**Ponderal index (grams/cm^3^)**	2.4 + 0.1	2.7 + 0.2*
**BMI (kg/m^2^)**	11.7 + 0.7	13.8 + 0.7*

* p < 0.05

IUGR = intrauterine growth restriction; BW = birth weight; WP50 = weight of 50th percentile; AC = arm circumference; HC = head circumference; BMI = body mass index.

There were no differences in maternal characteristics between the two groups, with regard to age, number of pregnancies, race and delivery type. Diseases like gestational hypertension and asthma were more frequent in group I; however, specific medications for these diseases were not used.

Male gender predominated among the newborns without IUGR (group II) and the gestational age was also significantly greater in group II. In accordance with the study design, newborns with IUGR presented birth weight, length, head circumference, BW/WP50 ratio, arm circumference/head circumference ratio (AC/HC), body mass index (BMI) and ponderal index (PI) that were smaller than those of the newborns without IUGR.

The arterial pressures increased during the first month of life. Systolic arterial pressure increased in a statistically significant manner from the first to the third day, from the third to the seventh day and from the seventh to the thirtieth day of life in both groups (Table 2). The systolic blood pressures did not differ significantly between the two groups at any time point. Moreover, there were no differences in mean and diastolic blood pressures between the groups.

**Table 2:** Systolic blood pressure evolution in the study groups of newborns

SBP (mmHg)	Group I with IUGR n = 35	Group II without IUGR n = 35
**1^st^day**	68.7 ± 6.8* (52.3-84.6)	69.7 ± 5.6* (58.6-78)
**3^rd^day**	75.1 ± 7.6 (59.3-90)	75.1 ± 4.9 (67.3-8.6)
**7^th^ day**	81.0 ±8.0 (68-95)	82.9 ± 4.9 (65.3-89)
**30^th^day**	89.3 ± 9.1 (66-100)	91.0 ± 5.9 (73-99.3)

SBP = systolic blood pressure; IUGR = intrauterine growth restriction.

Mean ± standard deviation (minimum-maximum)

* p < 0.05 SBP 1^st^ versus 3^rd^ versus 7^th^ versus 30^th^ day in each group.

The PRA levels did not differ between the newborns in the two groups (Table 3), at any of the times when measurements were made. However, there was a statistically significant reduction on the 30^th^ day of life in relation to the birth levels observed in both groups. PRA assays were not performed on all blood samples because there was insufficient material in 28 samples.

**Table 3:** Kidney weight and plasma renin activity in newborns

Renal variable	Group I with IUGR	Group II without IUGR
Kidney weight (grams)	5.9 ±1.6 * n = 35	9.6 ± 2.1 n = 35
(KW/BW) X 10^3^	2.6 ± 0.7	2.8 ± 0.6
PRA – birth (ng/ml/h)	10.5 ± 6.7† n = 27	8.3 ± 5.2† n = 27
PRA – 30^th^ day (ng/ml/h)	5.0 ± 3.7 n = 33	4.2 ± 2.8 n = 25

IUGR = intrauterine growth restriction; PRA = plasma renin activity.

* p < 0.001 kidney weight (newborns with IUGR versus newborns without IUGR); †p < 0.05 PRA (birth versus 30^th^ day), in each group; (KW/BW) x10^3^ = kidney weight/birth weight ratio x10^3^.

The newborns with IUGR presented kidney weights that were statistically smaller than in newborns without IUGR. However, when kidney weight was analyzed in relation to birth weight, there was no longer any difference (Table 3).

**Figure 1:**
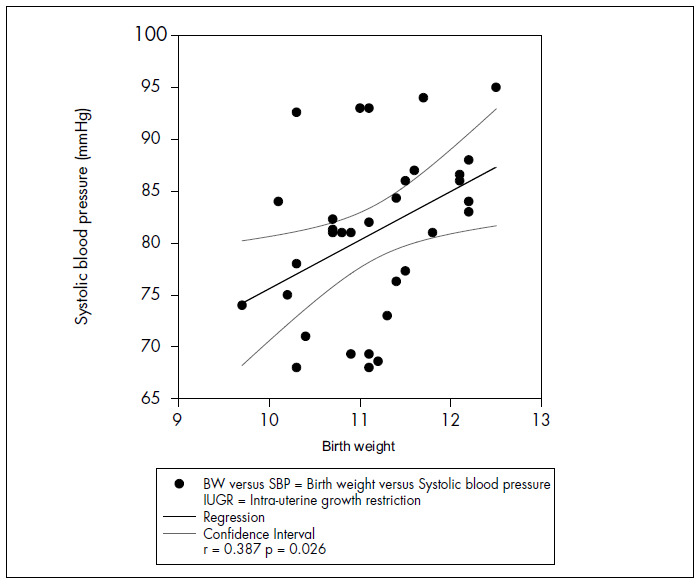
Correlation between birth weight and systolic blood pressure on the 7^th^ day of life (newborn with IUGR).

**Figure 2:**
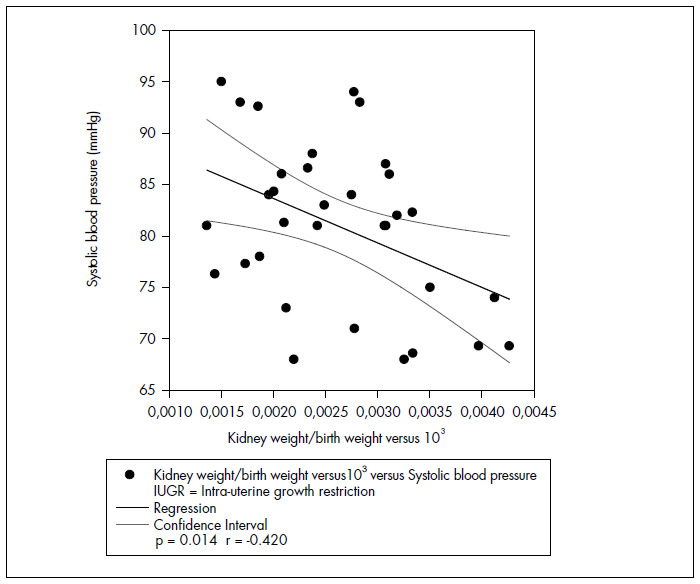
Correlation between kidney weight/birthweight versus 10^3^ and systolic blood pressure correlation on 7^th^ day of life (newborn with IUGR).

In newborns with IUGR, systolic blood pressure presented positive correlations with birth weight (r = 0.387 p = 0.026) and BMI (r = 0.412 p = 0.017) on the 7^th^ day of life (Figure 1). Positive correlations with birth weight (r = 0.440 p = 0.01) and birth length(r = 0.386 p = 0.026) were also seen on the 30^th^ day. There was an inverse correlation on the 7^th^ day between systolic arterial pressure and the kidney weight/birth weight ratio (Figure 2).

In neonates without IUGR, no correlations were observed between systolic blood pressure and birth weight, birth length, BMI or kidney weight/birth weight ratio.

## DISCUSSION

Adverse intrauterine conditions may program physiology and metabolism for the whole lifetime. Studies have produced evidence that the quality of intrauterine growth, as expressed through weight and body proportions at birth, could predict disease patterns in adults. At present, an inverse association between arterial hypertension and birth weight is recognized. This effect is present in childhood, less evident in adolescence and extends throughout adult life,^1^ although the importance of such an association has been questioned by some authors.^15^

The present study investigated the influence of IUGR on the evolution of systolic blood pressure levels over the first month of life. The natural history of blood pressure in full-term infants was well documented by the present authors in a previous study, using the same methodology.^16^ Although blood pressure increased significantly from the first to the 30^th^ day of life, it did not differ between the two groups. Therefore, newborns with or without IUGR presented similar blood pressure evolution during the neonatal period. Systolic blood pressure presented positive correlations with birth weight, length and BMI in the examinations performed on the 7^th^ and 30^th^ days of life in newborns with IUGR. In these analyses, blood pressure seemed to relate more to the physiological aspects of fetal growth, as represented by somatic development. Thus, heavier newborns presented higher blood pressure levels.

Hence, the inverse relationship between blood pressure and birth weight that has been recognized in childhood and adulthood was not yet present at this time. Therefore, these results concord with other published data^17-19^ that found a direct relationship between blood pressure and birth weight during the first days of life. The present study has added to these, by showing that such a relationship may extend to the entire neonatal period, on the basis of the results from the 30^th^ day of life. No correlation between blood pressure and birth weight was observed in the group without IUGR.

The mechanisms that control arterial pressure are not totally understood. In this context, the angiotensin-renin system can be highlighted because of its known role in the physiology of cardiovascular homeostasis.^20^ The levels of angiotensin-renin are extremely variable in studies of arterial hypertension. However, even at low levels, angiotensin-renin activity is present and is proven by the blood pressure response to angiotensin-converting enzyme inhibitors.^21^ In this light, more recent investigations have explored the possible role of angiotensin receptors (AT1 and AT2) in the origin and maintenance of arterial hypertension.^22^

No differences between PRA levels were observed in either group in this study. Langlay-Evans et al.^23^ also did not find any differences in PRA levels in hypertensive animals that had been subjected to protein restriction during the initial and intermediate gestational phases. However, PRA levels were higher when undernutrition was implemented at the end of pregnancy.

In the present study, PRA presented similar evolution in the two groups during the first month, with significant reduction in its levels from birth to the 30^th^ day of life. This result had already been described in the literature.^24-26^

Blood pressure levels did not correlate with PRA levels (at birth and on the 30^th^ day), in either group. These results concord with other authors’ results,^27,28^ in which no such correlation was also found.

On the other hand, the absence of correlation certainly does not rule out a role for the renin-angiotensin system in maintaining the blood pressure. It is likely that the relatively narrow arterial pressure limits that were observed acted in such a way that no correlation could have been demonstrated.

At first glance, the results presented demonstrate that the effect of IUGR on the evolution of systolic arterial pressure and PRA is not manifested during the neonatal period. On the other hand, experimental evidence has demonstrated that hypertensive animals present angiotensin II and PRA levels that differ little from control groups. Thus, it has been shown that future investigations on the physiology of angiotensin-converting enzymes and the expression of the AT1 and AT2 receptors would be a promising field in seeking to clarify these relationships.^29^

More and more evidence is emerging highlighting the important role of fetal programming in the development of adult hypertension. Animal studies and indirect evidence from human studies give support for the notion that low birth weight is associated with congenital deficits in the numbers of nephrons.^30-32^ The total number of nephrons is a biological variable that is defined before birth, and no new nephrons are formed after birth.^29^ Brenner et al.^33^ gathered impressive evidence in favor of the hypothesis that low nephron count is a risk factor for essential hypertension. They stated that the association between birth weight and arterial hypertension could reflect a correlation between the fetal growth pattern and the consequent number of nephrons.^33^

Various models for IUGR have demonstrated decreased nephron counts in rats.^5,6,34,35^ In these animal studies, reductions in kidney weight and volume persist throughout life.^36^ Seemingly, human pathological studies have found that asymmetric IUGR can exert a profound effect on kidney development. First, low birth weight full-term infants have lower kidney weight and lower glomerular profile density than do infants with greater birth weight.^37^ Second, the expected significant compensatory increase in nephron count is absent postnatally.^38^

Kidney size has been evaluated by ultrasound scan within the setting of growth restriction in human fetuses and children by several authors.^39,40^ Limitations caused by the use of ultrasound to assess kidney mass would be avoided by using other, more accurate methods such as magnetic resonance imaging, although this would be impractical.

Systolic blood pressure presented an inverse correlation with the kidney weight/birth weight ratio on the seventh day of life, among newborns with IUGR in the present study. This result may demonstrate that the effect of nutritional restriction on blood pressure during the neonatal period is manifested through kidney size. However, this association was not strong and did not persist to the 30^th^ day of life. No correlation between systolic blood pressure and the kidney weight/birth weight ratio was observed in the newborn group without IUGR.

## CONCLUSIONS

In summary, our findings demonstrate that the influence of intrauterine growth restriction on blood pressure is not manifested during the neonatal period. Birth weight correlated positively with systolic blood pressure in this population of full-term newborns du-ring the first month of life. Similar correlations were demonstrated for birth height and body mass index. The results from this study do not support the existence of relationships between kidney size at birth, plasma renin activity and systolic blood pressure. Such relationships will have to be confirmed in other prospective studies on larger populations of newborn before these results can be extrapolated to the general population.
